# Methylation of CENP-A/Cse4 on arginine 143 and lysine 131 regulates kinetochore stability in yeast

**DOI:** 10.1093/genetics/iyad028

**Published:** 2023-02-22

**Authors:** Tra My Tran Nguyen, Arno Munhoven, Anke Samel-Pommerencke, Rucha Kshirsagar, Alessandro Cuomo, Tiziana Bonaldi, Ann E Ehrenhofer-Murray

**Affiliations:** Institut für Biologie, Humboldt-Universität zu Berlin, 10099 Berlin, Germany; Institut für Biologie, Humboldt-Universität zu Berlin, 10099 Berlin, Germany; Institut für Biologie, Humboldt-Universität zu Berlin, 10099 Berlin, Germany; Institut für Biologie, Humboldt-Universität zu Berlin, 10099 Berlin, Germany; Department of Experimental Oncology, IEO European Institute of Oncology, IRCCS 20139 Milano, Italy; Department of Experimental Oncology, IEO European Institute of Oncology, IRCCS 20139 Milano, Italy; Department of Oncology and Hematology-Oncology, University of Milan, Milan 20122, Italy; Institut für Biologie, Humboldt-Universität zu Berlin, 10099 Berlin, Germany

**Keywords:** CENP-A, Cse4, Set2, Spc24, Spc25, Ndc80, Dsn1

## Abstract

Post-translational modifications on histones are well known to regulate chromatin structure and function, but much less information is available on modifications of the centromeric histone H3 variant and their effect at the kinetochore. Here, we report two modifications on the centromeric histone H3 variant CENP-A/Cse4 in the yeast *Saccharomyces cerevisiae*, methylation at arginine 143 (R143me) and lysine 131 (K131me), that affect centromere stability and kinetochore function. Both R143me and K131me lie in the core region of the centromeric nucleosome, near the entry/exit sites of the DNA from the nucleosome. Unexpectedly, mutation of Cse4-R143 (*cse4-R143A*) exacerbated the kinetochore defect of mutations in components of the NDC80 complex of the outer kinetochore (*spc25-1*) and the MIND complex (*dsn1-7*). The analysis of suppressor mutations of the *spc25-1 cse4-R143A* growth defect highlighted residues in Spc24, Ndc80, and Spc25 that localize to the tetramerization domain of the NDC80 complex and the Spc24-Spc25 stalk, suggesting that the mutations enhance interactions among NDC80 complex components and thus stabilize the complex. Furthermore, the Set2 histone methyltransferase inhibited kinetochore function in *spc25-1 cse4-R143A* cells, possibly by methylating Cse4-K131. Taken together, our data suggest that Cse4-R143 methylation and Cse4-K131 methylation affect the stability of the centromeric nucleosome, which is detrimental in the context of defective NDC80 tetramerization and can be compensated for by strengthening interactions among NDC80 complex components.

## Introduction

**Fig. 1.**
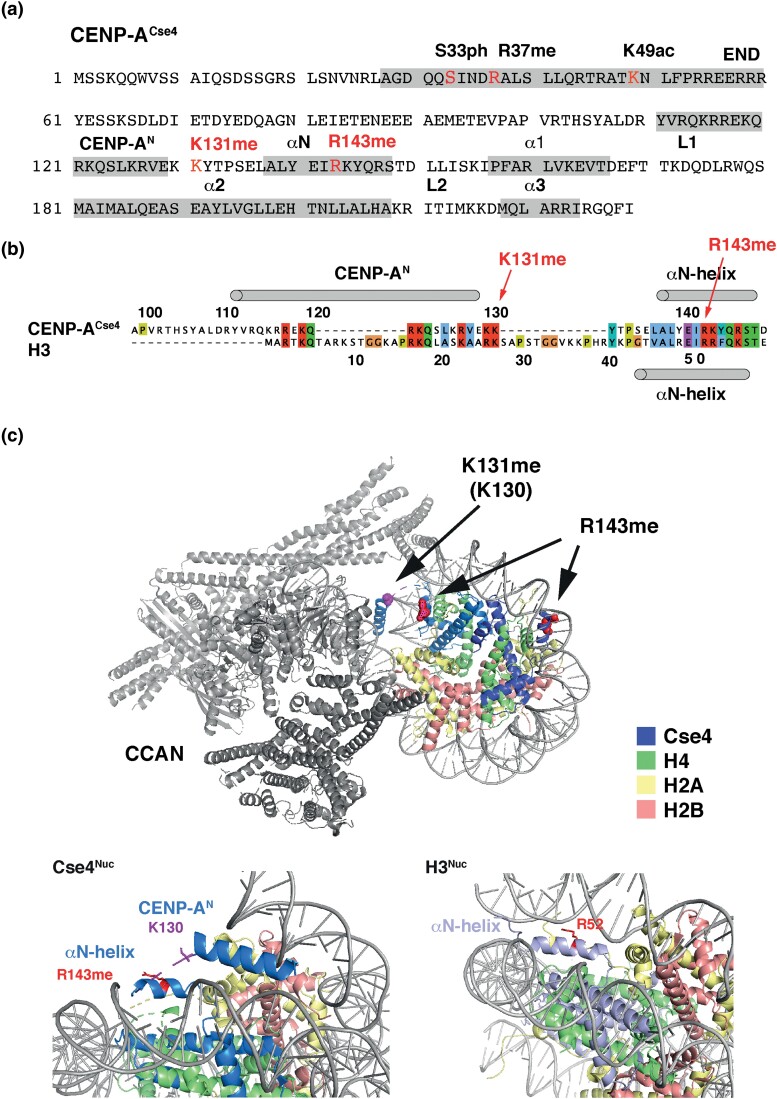
CENP-A^Cse4^ is methylated at arginine 143 and lysine 131. a) Overview of the amino acid sequence of Cse4. R143me and K131me sites are located in the α-N-helix and C-terminal to the Cenp-A^N^ helix, respectively. Cse4 is phosphorylated at S33 (S33ph), methylated at R37 (R37me), and acetylated at K49 (K49ac). b) R143 corresponds to R52 in canonical H3. Pairwise alignment of yeast H3 and Cse4 was generated with EMBOSS needle. c) Top, location of Cse4-R143 (red) and -K130 (magenta) in the context of CCAN-Cenp-A^Nuc^ (PDB 6QLD). K130 is shown, because K131 is not visible in the structure. Bottom, view of the α-N and Cenp-A^N^ helices and the location of R143 and K130 (as a proxy for K131) of CCAN-Cenp-A^Nuc^. The CCAN subunits were omitted for clarity.

Nucleosomes containing the centromeric histone H3 variant CENP-A mark the region on eukaryotic chromosomes where the kinetochore is assembled, which connects the centromeric DNA to the spindle microtubules [reviewed in (McAinsh and Marston 2022)]. Proper assembly of this macromolecular complex is crucial for correct chromosome segregation in mitosis and meiosis, and errors in this process can lead to aneuploidy. In higher eukaryotes, the centromeric chromatin is a broad domain (regional centromere) that is composed of CENP-A nucleosomes interspersed with canonical, H3-carrying nucleosomes (Allshire and Karpen 2008). The CENP-A nucleosomes are generally thought to be akin to conventional nucleosomes with the DNA wrapped left-handed around the histone octamer core, though a right-handed nucleosomal structure also has been proposed (Furuyama and Henikoff 2009; Diaz-Ingelmo *et al.* 2015). In contrast to higher eukaryotes, the yeast *Saccharomyces cerevisiae* has compact kinetochores that assemble on a single centromeric nucleosome (called a “point” centromere) and attach on their other end to a single microtubule (Furuyama and Biggins 2007). The fact that one nucleosome per chromosome is sufficient for faithful chromosome segregation in yeast may be attributed to the fact that Cse4 has a longer N-terminus than CENP-A homologs (135 amino-acids, Cse4N) that is essential for centromere function (Stoler *et al.* 1995; Keith *et al.* 1999). In earlier work, we and others showed that Cse4N interacts with two components of the constitutive centromere-associated network (CCAN) complex, Okp1 ^CENP-Q^ and Ame1 ^CENP-U^, and thus recruits CCAN to the centromere (Anedchenko *et al.* 2019; Fischböck-Halwachs *et al.* 2019). Furthermore, we identified post-translational modifications in Cse4N, the methylation of arginine 37 (R37me) and acetylation of lysine 49 (K49ac), as negative regulators of this interaction (Samel *et al.* 2012; Anedchenko *et al.* 2019). Also, we found that phosphorylation of S33 regulates the deposition of Cse4 at the centromere (Hoffmann *et al.* 2018) (Fig. 1a). Cse4 carries other phosphorylation sites (Boeckmann *et al.* 2013), and it is ubiquitinated and sumoylated (Hewawasam *et al.* 2010; Ranjitkar *et al.* 2010; Ohkuni *et al.* 2020).

The structure of CCAN bound to the Cse4-containing nucleosome (termed CCAN-Cenp-A^Nuc^) shows that CCAN assumes a “Y”-shaped structure that clasps onto the nucleosome (Yan *et al.* 2019). On its nucleosome-distal side, CCAN interacts with the MIND (Mis12/Mtw1) complex (Hornung *et al.* 2014), which itself forms an elongated, Y-shaped rod and contains the proteins Mtw1, Dsn1, Nsl1, and Nnf1 (Dimitrova *et al.* 2016). MIND interacts with the Okp1 and Ame1 components of CCAN as well as with the CENP-C homolog Mif2.

On the chromatin-distal side, MIND interacts with the main microtubule-binding entity of the kinetochore, the NDC80 complex (NDC80c) (Dimitrova *et al.* 2016). NDC80c is a heterotetrameric complex consisting of the proteins Ndc80, Nuf2, Spc24, and Spc25 that associate as two rod-like heterodimers (Ndc80/Nuf2, Spc24/Spc25) to form an elongated complex of ca. 600 Å in length (Janke *et al.* 2001; Wigge and Kilmartin 2001; Cheeseman *et al.* 2006; DeLuca *et al.* 2006; Wei *et al.* 2007). The two heterodimers each have an α-helical, coiled-coil shaft and globular domains at the ends (Wei *et al.* 2005; Ciferri *et al.* 2008), of which the Ndc80 and Nuf2 globular domains are microtubule-binding modules (Wei *et al.* 2007; Ciferri *et al.* 2008) and those of Spc24 and Spc25 bind components of the MIND complex in the inner kinetochore (Malvezzi *et al.* 2013; Hornung *et al.* 2014). The two α-helical coiled coils join end to end and form a tetramer junction (Valverde *et al.* 2016). The interactions of NDC80c with the microtubules and other kinetochore subcomplexes are tightly regulated by control mechanisms such as the spindle-assembly checkpoint (Santaguida and Musacchio 2009).

In this work, we describe two novel modifications on CENP-A/Cse4 in *S. cerevisiae*, the methylation of arginine 143 (R143me) and methylation of lysine 131 (K131me). These residues are located in the core domain of the centromeric nucleosome, close to the DNA duplex that is partially unwrapped in the centromeric nucleosome (Yan *et al.* 2019; Migl *et al.* 2020). We found that the absence of Cse4-R143me enhanced the centromeric defect of mutations in NDC80c (*spc25-1*) and MIND (*dsn1-7*). A screen for suppressors revealed intragenic mutations in *SPC25* as well as mutations in *SPC24*, *NDC80*, and *SET2*, which encode the histone methyltransferase for H3 K36 (Strahl *et al.* 2002). Genetic analysis suggests that Set2 acts at the kinetochore by methylating Cse4-K131. The location of the suppressor mutations in NDC80c indicates that *spc25-1* destabilizes the tetramerization junction and that the suppressor alleles strengthen interactions among the NDC80c components and thus suppress growth defects. Altogether, we propose that the absence of Cse4-R143me and -K131me affects the stability of the centromeric nucleosome, which is detrimental in the context of a compromised NDC80 complex.

## Materials and methods

### Yeast strains and plasmids

The *S. cerevisiae* strains and plasmids used in this study are listed in Supplementary Tables 1 and 2 (Supplementary File 1), respectively. Yeast was grown and manipulated according to standard procedures (Sherman 1991). Yeast was grown on full medium (YPD) and selective minimal plates (YM). Alleles of *cse4* were generated by site-directed mutagenesis in *CSE4*-bearing plasmids, and correct mutation was verified by sequence analysis. If plasmid-borne *cse4* alleles were tested, strains were constructed that carried *cse4Δ*, and cell viability was maintained with a *URA3-CSE4* plasmid (AEY2781, 5064). The *cse4* allele was introduced on a *HIS3*-carrying plasmid, and the *URA3-CSE4* plasmid was removed by counter-selection on medium containing 5-fluoro-orotic acid (5-FOA).

To test the effect of H3 K36A, strains were constructed that carried deletions of the cassettes *HHT1-HHF1* and *HHT2-HHF2* and whose viability was maintained by a *URA3*-marked plasmid carrying *HHT1-HHF1* (AEY7040, AEY7042, and AEY7045). *TRP1*-marked plasmids with *hht1-K36A-HHF1* or *HHT1-HHF1* as a control were transformed into the strains, and the *URA3*-marked *HHT1-HHF1* plasmid was removed by counter-selection on 5-FOA. The *hht1-K36A-HHF1* plasmid was obtained from a library of yeast strains carrying plasmid-borne histone mutations (Nakanishi *et al.* 2008) by amplifying the plasmid from the appropriate strain in *Escherichia coli*. The presence of H3 K36A was verified by sequence analysis.

Genomic integration of *cse4* alleles was performed by cloning the allele on a *URA3*-marked integrative vector and introducing it into the strain by integrative transformation followed by loop-out on 5-FOA. For genetic crosses, *cse4* was marked with *HisMX* by integration of the selection marker downstream of the open reading frame.

Plasmid loss was measured in a wt (AEY1), *cse4-R143A* (AEY6831), *spc25-1* (AEY4924), and *spc25-1 cse4-R143A* (AEY6838) strain carrying a CEN6-*TRP1* plasmid (pAE264) as previously described (McNally and Rine 1991). For statistical analysis of biological triplicates, a one-sided *t*-test was employed. For FACS analysis, strains were grown in YPD at 23°C and shifted for 3 h to 30°C. Samples of 0.2 OD were harvested in mid-exponential phase and processed as previously described (Anedchenko *et al.* 2019).

### Mass spectrometric analysis of Cse4

Purification and analysis of 3xHA-tagged Cse4 from yeast cells were performed as described (Samel *et al.* 2012) (see Supplementary File 1 for details).

### Screen for suppressors of *spc25-1 cse4-R143A* and whole-genome sequence analysis

The *spc25-1 cse4-R143A* strain (AEY6838) was spread at a density of 3 × 10^6^ cells on YPD plates. Plates were UV-irradiated with 3000 µJ/cm^2^ (wave length 254 nm) in a Stratalinker 2400 UV Crosslinker and subsequently incubated for 4–5 days at 34°C in the dark. This UV dosage gives a survival rate of ∼60% when cells are grown at 23°C. Surviving colonies were re-streaked and grown on YPD at 23°C. Growth at different temperatures was determined by plating serial dilutions of the strains on YPD and incubating the plates at different temperatures for 3 days.

For sequencing of the *spc25* and *cse4* alleles of the suppressor strains, the genes were PCR-amplified, and the PCR products were subjected to DNA sequencing. In *spc25-1*, we observed a silent mutation (Spc25-Q38, CAA to CAG) in addition to the reported L25P exchange (Wigge and Kilmartin 2001).

For whole-genome sequence analysis (WGS), strains were grown individually, and groups of five strains were mixed in equal quantities of cells (OD_600_ equivalents). DNA was extracted from 10 such pools, sequencing libraries were prepared, and sequencing was conducted on an Illumina NovaSeq6000 instrument (paired-end sequencing, 150 bp) to obtain ∼5 × 10^6^ read pairs per pool. Reads were aligned to the reference genome (SacCer3) using Bowtie2, and single-nucleotide polymorphisms (SNPs) were called using deepSNV (Gerstung *et al.* 2012). The presence of suppressor mutations in individual strains was verified by sequence analysis of PCR-amplified alleles. Sequencing reads were deposited in the National Center for Biotechnology Information (NCBI) Sequence Read Archive (SRA) at http://www.ncbi.nlm.nih.gov/sra under accession no. PRJNA933081.

To verify whether mutations are causative for the suppression of *spc25-1 cse4-R143A*, the segregation of temperature resistance with the mutation was determined in backcrosses with a *spc25-1 cse4-R143A* parental strain and sequencing of PCR-amplified alleles. Furthermore, for *spc24-D37A*, *spc24-S59L*, and *ndc80-L681W*, five temperature-resistant and five temperature-sensitive segregants from the backcross were pooled and subjected to WGS. All temperature-resistant and no temperature-sensitive segregants carried the respective suppressor mutation.

## Results

### CENP-A^Cse4^ is methylated on arginine 143 and lysine 131

Here, we report the identification of two novel modifications in residues of Cse4 that are within the core region of the centromeric nucleosome, rather than in the N-terminus. In purifications of Cse4 from *S. cerevisiae* cells that were analyzed by gel-enhanced liquid chromatography mass spectrometry (GeLCMS) (Samel *et al.* 2012), we identified mono-methylation of arginine 143 (R143me1) and mono-methylation of lysine 131 (K131me1) (Fig. 1a, Supplementary Fig. 1 in Supplementary File 1). Specifically, collision-induced dissociation fragmentation analysis of trypsin-digested Cse4 identified the peptide 131-KYTPSELALYEIR-143 as monomethylated. Inspection of the MS/MS spectrum of the parent ion allowed us to uniquely assign the methylation site to R143 (Supplementary Fig. 1a in Supplementary File 1). Furthermore, fragmentation analysis identified the same peptide as being monomethylated at K131 (Supplementary Fig. 1b in Supplementary File 1). Of note, Cse4-K131 can be ubiquitinated in vitro by the E3 ubiquitin ligase Psh1, but whether this occurs in vivo was not determined (Hewawasam *et al.* 2010).

It is interesting to consider the position of these modifications in the context of the CCAN-bound centromeric nucleosome (CCAN-Cenp-A^Nuc^) (Yan *et al.* 2019). The R143 methylation site corresponds to R52 in histone H3 (Fig. 1b) and lies within the α-N helix of Cse4 that is in close proximity to the DNA of the Cse4 nucleosome. However, in contrast to the canonical H3 nucleosome, where the corresponding helix is wedged between two DNA helices (Fig. 1c) (Luger *et al.* 1997), the DNA in CCAN-Cenp-A^Nuc^ is more loosely wrapped (Migl *et al.* 2020), and the α-N helix of Cse4 is not wedged between the helices. Rather, a helix N-terminal to α-N in one of the Cse4 units of the nucleosome (aa 111–129, termed Cenp-A^N^) is inserted between the unwrapped DNA duplex and DNA gyre (Yan *et al.* 2019). Notably, the K131 methylation site lies two amino acid C-terminal to this helix, but only K130 (Fig. 1b), not K131 itself, is visible in the CCAN-Cenp-A^Nuc^ structure. The alignment of Cse4 with H3 suggests that Cse4-131 is equivalent to K27 in H3. It should, however, be noted that H3 does not contain an equivalent α-helix, and this must therefore be interpreted with caution. Due to their proximity to the DNA at the entry/exit site of the DNA in the nucleosome, both R143 and K131 methylation can be hypothesized to affect the interaction of CENP-A with the DNA in a left-handed nucleosome. How a right-handed nucleosome configuration would be affected by these modifications remains to be seen (Furuyama and Henikoff 2009).

### Cse4-R143A causes a synthetic growth defect with *spc25-1* and *dsn1-7*

**Fig. 2.**
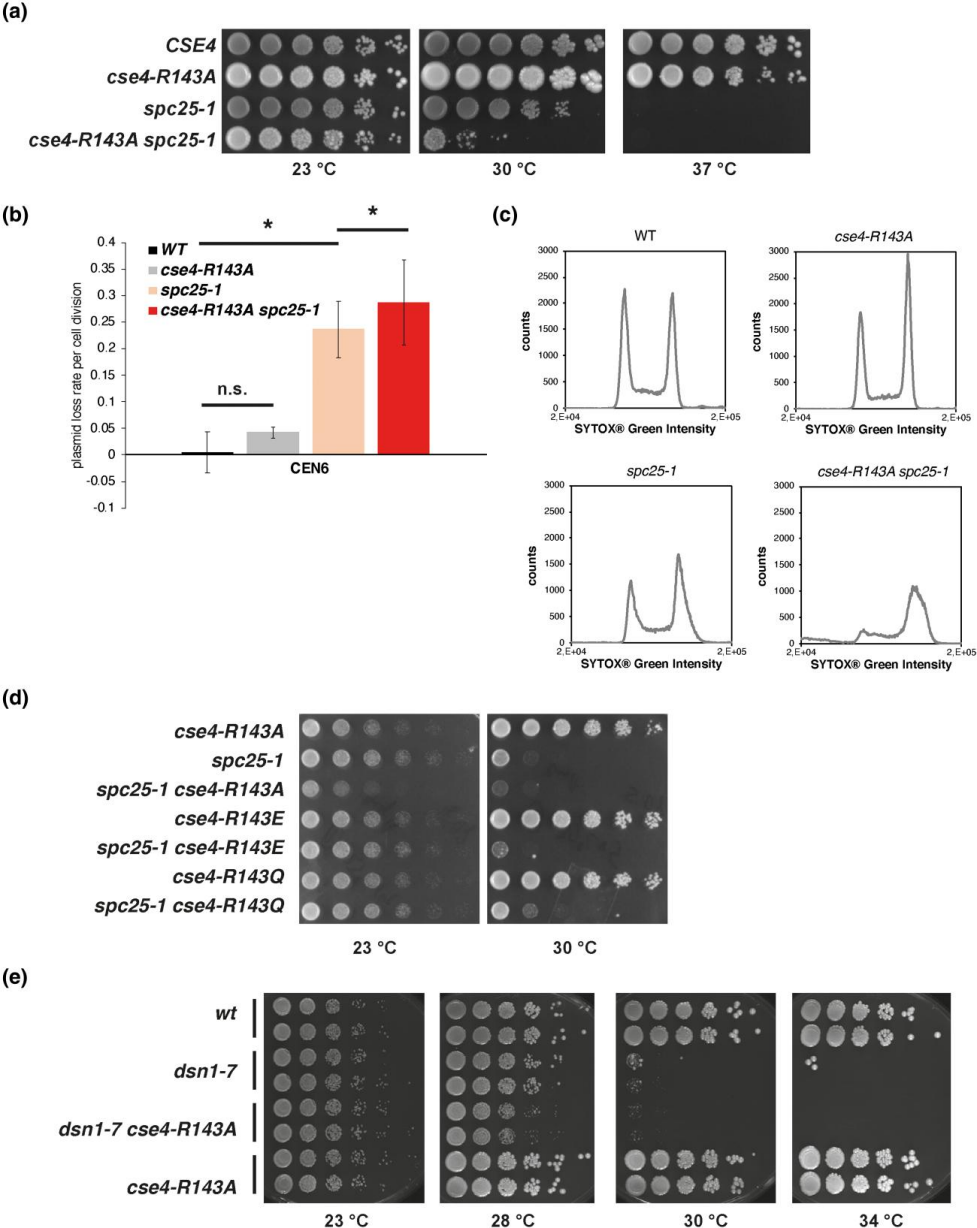
The absence of Cse4-R143 methylation enhances the centromeric defect of *spc25-1* and *dsn1-7*. a) Mutation of Cse4-R143 to alanine caused slow growth in the *spc25-1* background. Strains with the indicated genotypes were serially diluted and spotted on YPD medium, and plates were incubated for 3 days and the indicated temperatures. b) *cse4-R143A* enhanced the plasmid maintenance defect of *spc25-1*. Error bars give SD of three independent experiments. *Significant difference, *P* < 0.05. n.s., not significant. c) *spc25-1 cse4-R143A* cells arrested at the G2/M phase of the cell cycle at the restrictive temperature. Cells were grown to early logarithmic phase at 23°C and shifted to 30°C for 3 h. DNA content as measured by FACS analysis is shown. d) *cse4-R143E* and *-R143Q* enhanced the growth defect of *spc25-1*. Serial dilutions of *cse4Δ* strains with the *cse4* alleles on a plasmid were spotted on YPD plates and incubated for 2 days at the indicated temperatures. e) cse4-R143A enhanced the growth defect of *dsn1-7*. Serial dilutions of the indicated strains were spotted on YPD plates and growth for 2 days at the indicated temperatures.

**Fig. 3.**
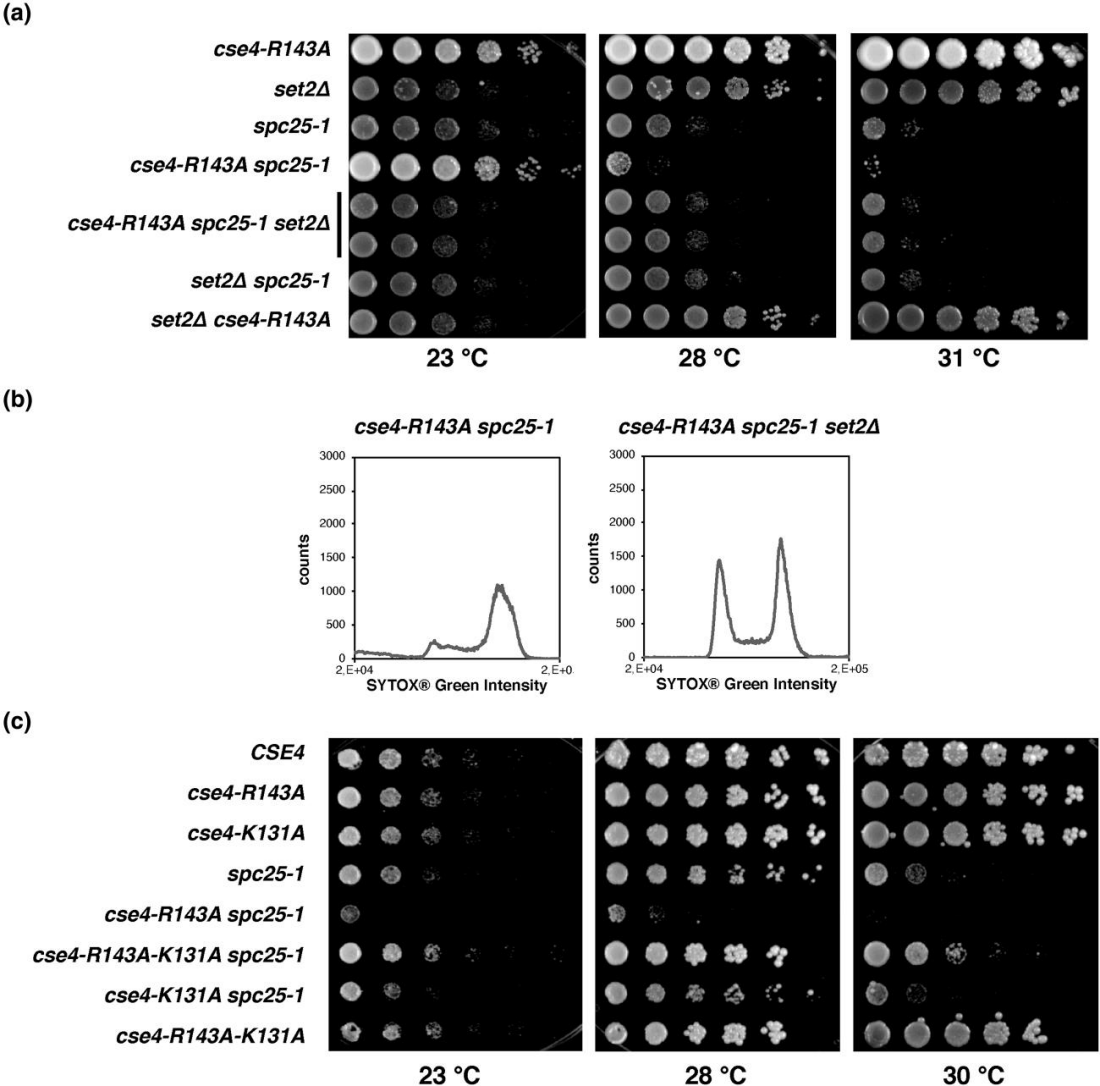
The deletion of *SET2* partially suppressed the centromeric defect of *cse4-R143A spc25-1*. a) *set2Δ* suppressed the temperature-sensitive growth defect of *spc25-1 cse4-R143A*, but not *spc25-1* alone. Cell growth was assessed as in Fig. 2a. b) *set2Δ* suppressed the cell-cycle defect of *spc25-1 cse4-R143A*. FACS was conducted as in Fig. 2c. The experiment was carried out simultaneously with that presented in Fig. 2c. For further controls, see Supplementary Fig. 3a in Supplementary File 1. c) *cse4-K131A* partially suppressed the growth defect of *spc25-1 cse4-R143A*. Analysis was performed with plasmid-borne *cse4* alleles as in Fig. 2d.

We next sought to determine how the absence of methylation on Cse4-R143 and -K131 affects Cse4 function at the centromere. For this, strains were created that carried alleles of *CSE4* in which these residues were replaced by alanine (*cse4-R143A*, *cse4-K131A*). Neither mutation caused an appreciable growth defect or temperature sensitivity (Figs. 2a, e, and 3c), showing that these modifications as such are not required for cell viability.

We subsequently tested whether *cse4-R143A* causes synthetic growth defects when combined with mutations in kinetochore components that reduce centromeric function (see below for *cse4-K131A*). Interestingly, *cse4-R143A* strongly enhanced the growth defect of cells carrying a mutation in the NDC80 component Spc25 (*spc25-1*, Fig. 2a) (Wigge and Kilmartin 2001). Specifically, the *spc25-1* allele alone causes slightly reduced growth at 30°C and is unable to grow at 37°C, and the additional *cse4-R143A* mutation strongly enhanced the growth defect at 30°C. This result indicates that Cse4-R143me becomes important when Spc25 function is compromised.

The growth defect of *spc25-1 cse4-R143A* suggested that the chromosome segregation defect of *spc25-1* is enhanced in these cells. To test this, we examined the stability of minichromosomes (plasmids) in cells that were wild type (wt) or carried one or both mutations. As expected, *spc25-1*, but not *cse4-R143A* alone, increased the plasmid loss rate, indicating a compromised centromere function of the plasmid. Importantly, *cse4-R143A* further exacerbated the plasmid maintenance defect of *spc25-1*, thus underscoring the notion that it enhanced the chromosome segregation defect (Fig. 2b). This indicated that Cse4-R143 methylation affects kinetochore function when Spc25 function is compromised.

We furthermore investigated how *cse4-R143A* affected cell-cycle progression in *spc25-1* cells. Incubation of *spc25-1* cells at the semi-permissive temperature of 30°C for 3 hours had little effect on the proportions of cells with 1n or 2n DNA content, as determined by measuring the DNA content by FACS analysis (Fig. 2c). Importantly, *spc25-1 cse4-R143A* cells were arrested with a 2n DNA content under this condition, indicating that Cse4-R143A caused a defect in centromere function and chromosome segregation at the G2/M boundary.

The phenotypes observed above for *cse4-R143A* point towards a function for R143 in kinetochore stability. We were therefore interested to see whether other amino-acid substitutions can enhance the effect or can cause a phenotype of the Cse4-R143 substitution alone, without *spc25-1*. A replacement of R143 with the negatively charged glutamate (*cse4-R143E*) caused a similar phenotype as *cse4-R143A* in that the mutation alone had no effect on viability but enhanced the defect of *spc25-1*. *cse4-R143Q*, a replacement with a neutral charge, caused a slightly less pronounced defect in *spc25-1* than the other two *cse4* alleles (Fig. 2d). Thus, at least these, and perhaps all mutations in Cse4-R143, only cause a defect in the context of a compromised kinetochore, as is the case in the *spc25-1* background.

The genetic interaction of *cse4-R143A* with *spc25-1* was surprising, because Spc25 is a component of NDC80c (Wigge and Kilmartin 2001), which interacts on its chromatin-proximal side with the MIND complex and connects the inner kinetochore to the Dam ring and the microtubule. Spc25 thus is thought to be spatially distant from the Cse4-containing nucleosome (McAinsh and Marston 2022). A comprehensive genetic analysis of *cse4-R143A* with mutations in genes encoding kinetochore components revealed only one other mutation to have a genetic interaction with *cse4-R143A*, *dsn1-7* (Nekrasov *et al.* 2003), in that the *cse4-R143A dsn1-7* double mutants showed an enhanced temperature-sensitive growth defect (Fig. 2e). Dsn1 is a component of the MIND complex (De Wulf *et al.* 2003), which links NDC80c to CCAN in the inner kinetochore. Notably, *cse4-R143A* did not enhance growth defects of mutations in many other components of the inner and outer kinetochore (Table 1). Perhaps most surprising was the absence of phenotypes with mutations in other NDC80 components, notably Spc24, the most intimate physical interactor of Spc25 (Janke *et al.* 2001; Wei *et al.* 2005; Ciferri *et al.* 2008; Hornung *et al.* 2014) (see Discussion). Cse4-R143A also did not display genetic interactions with other MIND, CCAN, or CBF3 complex components, or with Cbf1. This was in striking contrast to our earlier analysis of mutations in the Cse4 N-terminus, which showed highly selective genetic interactions with components of CCAN and with Cbf1, thus highlighting the functional difference of Cse4-R143 methylation with R37 methylation, K49 acetylation, and S33 phosphorylation (Samel *et al.* 2012; Hoffmann *et al.* 2018; Anedchenko *et al.* 2019).

**Table 1.** Synthetic genetic interactions of *cse4-R143A* with mutations in genes encoding kinetochore components*^a^*.

Kinetochore component/complex	Allele	Synthetic phenotype with *cse4-R143A*
Ctf19	*cnn1Δ*	*—*
	*wip1Δ*	*—*
	*iml3Δ*	*—*
	*chl4Δ*	*—*
	*ctf3Δ*	*—*
CBF3	*csm3Δ*	*—*
	*ctf13-30*	*—*
Mif2	*mif2-3*	*—*
Other	*cbf1Δ*	*—*
COMA	*okp1-5*	*—*
	*ame1-4*	*—*
	*ctf19Δ*	*—*
	*mcm21Δ*	*—*
Ndc80	*ndc80-1*	*—*
	*nuf2-61*	*—*
	*spc25-1*	Growth defect
	*spc24-1*	*—*
Mtw1	*mtw1-11*	*—*
	*dsn1-7*	Growth defect
	*nsl1-5*	*—*
	*nnf1-77*	*—*
Spc105	*spc105-1*	*—*

Additional phenotype caused by *cse4-R143A* in combination with the indicated allele of the gene encoding the respective kinetochore component. —, no additional phenotype observed.

### Genetic screen for suppressors of the temperature sensitivity of *spc25-1 cse4-R143A*

The synthetic genetic interactions of *cse4-R143A* with *spc25-1* and *dsn1-7* were surprising given the assumed spatial distance between the respective proteins within the architecture of the kinetochore, raising the question of the mechanistic basis of the defect. To investigate this, we characterized intra- and extragenic suppressors of the temperature-sensitive growth defect of *spc25-1 cse4-R143A*. We surmised that such mutations would provide information about the defect of *spc25-1*, and thus by inference, also of *cse4-R143A*. The expectation was that some suppressors would specifically suppress *spc25-1 cse4-R143A*, whereas others would suppress *spc25-1* alone. Operationally, *spc25-1 cse4-R143A* cells were UV-mutagenized, plated, and incubated at the semi-restrictive temperature of 34°C for 4–5 days. This temperature was chosen in the hope of reducing the likelihood of isolating revertants of *spc25-1*, which carries a point mutation [CTG to CCG, L25P (Wigge and Kilmartin 2001)] and was therefore expected to easily revert back to wild type.

A total of 148 mutants were isolated that showed different degrees of suppression of the temperature-sensitive growth defect, ranging from poor growth at 34°C to moderate growth at 37°C. To distinguish between revertants and intragenic suppressors, the *spc25* alleles of these strains were PCR-amplified and sequenced. Indeed, 29 of the 148 strains showed a reversion of the *spc25-1* mutation (see Supplementary Fig. 2 in Supplementary File 1 for a flow chart of the analysis). Interestingly, 66 strains carried an intragenic suppressor mutation. They will be described below. The remaining 53 suppressors still carried the original *spc25-1* allele (*spc25-L25P*). They were further investigated for whether they carried a mutation of the *cse4-R143A* allele, since reversion or other mutations in *CSE4* in principle might also suppress the temperature sensitivity of the double mutant. Reversion is expected to be rare, because the mutation carries two base substitutions (CGA to GCA). The analysis showed that all 53 remaining suppressors still carried *cse4-R143A*.

Next, the 50 strongest suppressors were subjected to whole-genome sequencing (WGS) in order to identify the causative mutation. For economic reasons, they were grouped in 10 pools of five strains depending on their strength of growth at elevated temperatures, and the pools were subjected to WGS and SNP analysis. Here, we describe mutations in *SET2*, *SPC24*, and *NDC80* as causative for the suppression, which accounts for 25 of the 50 mutants (Supplementary Fig. 2 in Supplementary File 1). The analysis of other mutants will be described elsewhere.

### *set2Δ* suppresses the centromere defect of *spc25-1 cse4-R143A*

Interestingly, one of the *spc25-1 cse4-R143A* suppressor mutants was found to carry a mutation in *SET2* (*set2-P253S*, CCA to TCA). Set2 is a histone lysine methyltransferase that associates with the elongating RNA polymerase II and methylates H3 K36 during transcription elongation (Krogan *et al.* 2003; Xiao *et al.* 2003). This serves to recruit the Rpd3S histone deacetylase complex, which removes acetylation marks on chromatin in the open reading frames (ORFs) after transcription and thus suppresses intragenic transcription emanating from cryptic transcription start sites within the ORF (Carrozza *et al.* 2005; Keogh *et al.* 2005). More recently, Set2 has been described to methylate H3 K37, and this protects the genome from spurious DNA replication (Santos-Rosa *et al.* 2021).

The function of Set2 as a chromatin modifier made it an interesting candidate to pursue. We tested whether the deletion of *SET2* (*set2Δ*) caused the same phenotype as the point mutation isolated in the screen. Indeed, *set2Δ* suppressed the growth defect of *spc25-1 cse4-R143A* at semi-permissive temperatures, and the growth defect of *spc25-1* alone was not suppressed (Fig. 3a). Furthermore, the arrest of *spc25-1 cse4-R143A* cells in G2/M at the semi-permissive temperature was suppressed by *set2Δ* (Fig. 3b, Supplementary Fig. 3a in Supplementary File 1). These results indicated that the absence of Set2 function alleviated the centromeric defect caused by Cse4-R143A in *spc25-1* strains.

Since Set2 methylates H3 K36 (Strahl *et al.* 2002; Krogan *et al.* 2003; Li *et al.* 2003), a first hypothesis was that the suppression of *spc25-1 cse4-R143A* is the result of the absence of H3 K36me and concomitant changes in transcription, for instance of genes encoding kinetochore components. Of note, published transcriptome datasets did not reveal changes in the transcription of kinetochore components in mitotic cells in *set2Δ* (Venkatesh *et al.* 2016), though Set2 contributes to the repression of *NDC80* transcription during meiosis (Chen *et al.* 2017; Chia *et al.* 2017). To further test a possible effect of H3 K36 methylation, we constructed *spc25-1 cse4-R143A* strains in which H3 K36 was exchanged for alanine (H3 K36A), in order to imitate the unmethylated state. We observed that H3 K36A improved the growth of both *spc25-1* and *spc25-1 cse4-R143A* at the semi-permissive temperature (Supplementary Fig. 3b in Supplementary File 1). This effect therefore was distinct from that of *set2Δ*, which only suppressed *spc25-1 cse4-R143A*, arguing against H3 K36 methylation as the causative mechanism for the suppression.

Since we have identified Cse4-K131 to be methylated (Supplementary Fig. 1b in Supplementary File 1), we considered the possibility that Set2 could methylate K131 and that the absence of this methylation would specifically suppress the growth defect of *spc25-1 cse4-R143A*. To this end, we constructed an allele of *CSE4* in which both R143 and K131 were replaced by alanine (*cse4-R143A-K131A*) and tested how this affected the temperature sensitivity of *spc25-1*. Significantly, *spc25-1 cse4-R143A-K131A* strains showed improved growth compared to *spc25-1 cse4-R143A*. Furthermore, *cse4-K131A* alone in *spc25-1* did not improve growth (Fig. 3c). Thus, with respect to its suppression phenotypes, *cse4-K131A* resembles the effect of *set2Δ*. A speculative interpretation of this result therefore is that Set2 methylates Cse4-K131 and that this methylation negatively impacts centromeric function. Notably, this was the only phenotype observed for *cse4-K131A*, and this mutation showed no synthetic defects with several mutations in kinetochore components (Supplementary Table 3 in Supplementary File 1). Alternatively, based on the observation that Set2 regulates a non-coding transcript that controls *NDC80* expression in meiosis (Chen *et al.* 2017; Chia *et al.* 2017), it may similarly affect mitotic centromere function by regulating an as yet unknown non-coding transcript.

### Suppressor mutations of *spc25-1 cse4-R143A* are located in the tetramer junction and the Spc24-Ndc80 coiled coil of the NDC80 complex

**Fig. 4.**
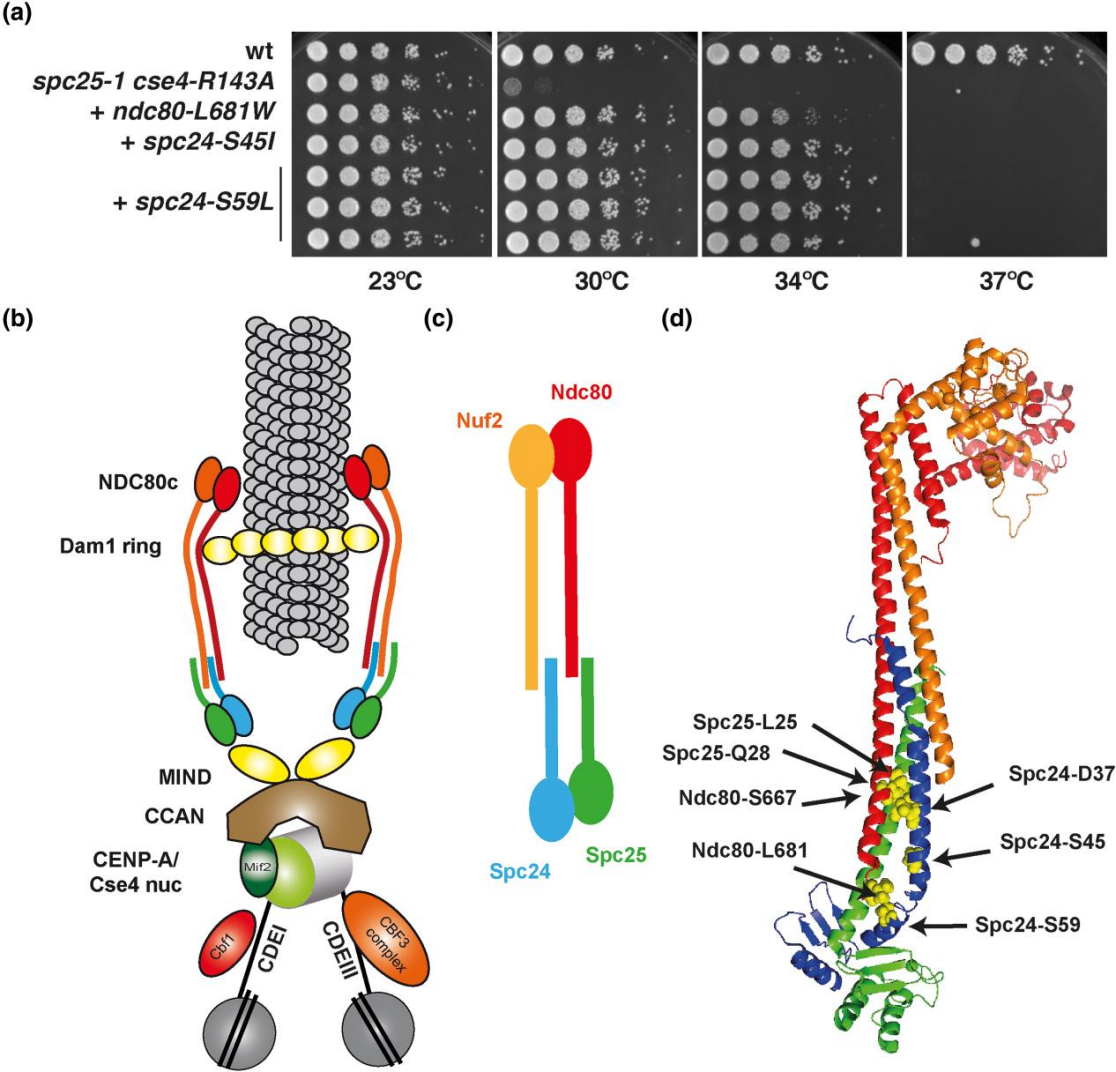
Suppressor mutations of *spc25-1 cse4-R143A* are located in the tetramer junction and the Spc24-Ndc80 coiled coil of the NDC80 complex. a) Suppression of the growth defect of *spc25-1 cse4-R143A* by mutations in *NDC80* and *SPC24*. Serial dilutions of the strains were spotted on full medium and grown for 3 days at the indicated temperatures. b) Schematic of the *S. cerevisiae* kinetochore. c) Schematic of NDC80c illustrating the subcomplexes of Nuf2 with Ndc80 and Spc24 with Spc25. d) Localization of residues mutated in second-site suppressors of the *spc25-1 cse4-R143A* temperature-sensitive growth defect in the structure of the NDC80c^e-dwarf^ structure (PDB 5TD8, (Valverde *et al.* 2016)). Color schematic of proteins as in b). The respective residues are shown as yellow spheres. The suppressor mutations localize to the tetramerization junction and the Spc24-Spc24 stalk. Image generated using PyMol.

In the screen for suppressors of the temperature sensitivity of *spc25-1 cse4-R143A*, we isolated 66 second-site mutations in *SPC25* representing 9 different alleles (Table 2). Furthermore, 24 isolates carried mutations in *SPC24* (6 alleles) or *NDC80* (3 alleles, Table 3; see Fig. 4a for temperature resistance of selected mutants). Like Spc25, Spc24 and Ndc80 are components of NDC80c (Fig. 4b) (Wigge and Kilmartin 2001; De Wulf *et al.* 2003), and it therefore seems most likely that these mutations are causative for the suppression of the *spc25-1 cse4-R143A* temperature sensitivity. To test this, the strains with the mutations *spc24-D37A*, *spc24-S59L*, and *ndc80-L681W* were backcrossed to a *spc25-1 cse4-R143A* parent strain, and five temperature-resistant and five temperature-sensitive segregants (as a control) were pooled and subjected to WGS. In all three cases, the temperature-resistant but not the temperature-sensitive segregants carried the respective *spc24* or *ndc80* mutation, indicating that they indeed were responsible for the suppression. Furthermore, backcrosses of *ndc80-L681W*, *spc24-S45I*, and *spc24-S59L* to a *spc25-1* and a wt strain showed (1) that these mutations suppressed the temperature sensitivity of *spc25-1* alone (without *cse4-R143A*) and (2) that the mutations themselves did not cause a growth defect.

**Table 2.** Intragenic suppressors of *spc25-1*.

Allele	Number of isolates
*spc25-L25P-T81I (Hs L106)*	40
*spc25-L25S*	9
*spc25-L25Q*	5
*spc25-L25A*	4
*spc25-L25P-Q28L*	3
*spc25-L25R*	2
*spc25-L25P-G117D (Hs Q132)*	1
*spc25-L25P-L123S (K133 – S134)*	1
*spc25-L25V*	1

**Table 3.** Alleles of *SPC24* and *NDC80* that suppress *spc25-1 cse4-R143A*.

Allele	Number of isolates
*spc24-D37A*	1
*spc24-D37Y*	3
*spc24-S59L*	12
*spc24-S45I*	1
*spc24-S134F*	3
*spc24-S134Y*	1
*ndc80-S667A*	1
*ndc80-S667C*	1
*ndc80-L681W*	1

The location of these mutations, as well as of the *spc25* intragenic suppressors, in the respective proteins provides interesting insights in the context of the structure of NDC80c. NDC80c is a tetramer with two heterodimeric subcomplexes (Ndc80/Nuf2 and Spc24/Spc25) that connect end to end via a tetramerization domain (Fig. 4c) (Janke *et al.* 2001; Wigge and Kilmartin 2001; Cheeseman *et al.* 2006; DeLuca *et al.* 2006; Wei *et al.* 2007). The structure of a shortened “dwarf” version of yeast NDC80c shows two intertwined α-helical coiled coils with contacts between the heterodimers (Fig. 4d) (Valverde *et al.* 2016). The mutated residue in the *spc25-1* allele, L25, is located in a bundle of three α-helices formed by Spc25, Spc24, and Ndc80 within the junction of NDC80c, and the mutation to proline can be hypothesized to disrupt the α-helix and thus to disturb the tetramerization of NDC80c. Interestingly, positions Spc24-D37, S45, and Ndc80-S667, which are highlighted by the respective suppressor alleles, are all located within the three-helix bundle structure formed by Spc24 and Spc25 with Ndc80 (Fig. 4d). This suggests that the mutations strengthen the interaction with the mutant Spc25-L25P protein and thus are able to suppress the temperature-sensitive growth defect. Furthermore, Spc25-Q28, which is highlighted by an intragenic suppressor, is also located in this region, suggesting that the mutation Q28L improves interaction to the closest protein, Ndc80. Also, the residues Spc24-S59 and Ndc80-L681 lie within the coiled-coil region formed by the two proteins, again indicating that the suppressor mutations stabilize the interaction between the two proteins and thus suppress the kinetochore defect of *spc25-L25P*.

It is noteworthy that among all extragenic suppressors isolated here, we did not find any mutations in *NUF2*, which encodes the fourth component of NDC80c. This may be due to the fact that *spc25-1* does not directly affect the interaction to Nuf2 and such suppressor mutations in *NUF2* hence may not exist.

Two residues highlighted by the suppressors, Spc25-G117 and -L123, are not present in the “dwarf” NDC80c structure (Valverde *et al.* 2016). Pairwise alignment shows that Spc25-G117 is equivalent to Q132 of human Spc25, and L123 lies in a yeast-specific short loop that is located between K133 and S134 of the human protein (Supplementary Fig. 4a in Supplementary File 1). These residues lie within the coiled-coil region of Spc25 and Spc24 in the Ndc80^ΔN-bonsai^ structure (Supplementary Fig. 4b in Supplementary File 1) (Ciferri *et al.* 2008). As above, the location of the respective suppressor mutations in the α-helical stalk formed by Spc25 and Spc24 suggests that they improve the binding between the two proteins and thus can compensate for the *spc25-L25P* mutation. Spc24-S134 is not present in either structure.

Our interpretation of the suppressor alleles that mutated Spc25-L25P to another amino acid is that these mutations (1) abrogate the helix-breaking proline and/or (2) cause an enhanced interaction with Spc24 and Ndc80.

Taken together, the analysis of the *spc25-1 cse4-R143A* suppressors suggests that they enhance kinetochore function by strengthening protein–protein interactions among the components of NDC80c. By inference, this suggests that the absence of Cse4-R143 methylation contributes to kinetochore failure, possibly by affecting the centromeric nucleosome, and that this can be compensated for by improving the stability of NDC80c.

## Discussion

Post-translational modifications on histones are important regulators of chromatin function. Here, we describe two new modifications on the centromeric histone H3 variant CENP-A/Cse4 in *S. cerevisiae*, methylation of Cse4-R143 and methylation of Cse4-K131. Mutation of Cse4-R143 caused a synthetic growth defect and enhanced minichromosome loss and a chromosome segregation defect and a defect in G2/M phase transition when the function of Spc25, a component of NDC80c, was compromised. Furthermore, *cse4-R143A* caused a defect when combined with a mutation in *DSN1*, which encodes a component of the MIND complex (Euskirchen 2002; Nekrasov *et al.* 2003). We found several mutations in *SPC24*, *NDC80*, and *SPC25* itself to suppress the defect of *spc25-1 cse4-R143A*, and their location within the structure of NDC80c suggests that they act by strengthening protein–protein interactions among these three components. This in turn indicates that the centromeric defect of *spc25-1 cse4-R143A* cells is the result of enhanced kinetochore failure when Cse4-R143A is mutated, possibly because it affects the centromeric nucleosome. Furthermore, the Set2 methyltransferase had a negative function at the centromere, because *set2Δ* partially suppressed the *spc25-1 cse4-R143A* defect, possibly via methylation of Cse4-K131. Of note, the defects caused by the absence of Cse4-R143 or -K131 methylation were markedly different from those of the PTMs in Cse4N, which inhibit the interaction between Cse4N and the Okp1 ^CENP-U^ and Ame1 ^CENP-Q^ components of the CCAN complex (Anedchenko *et al.* 2019).

It was surprising that *cse4-R143A* showed a specific defect with a mutation in *SPC25*, since Spc25 is located at the outer kinetochore and therefore is thought to be physically distant from the Cse4 nucleosome within the architecture of the kinetochore. From a formal genetic point of view, this genetic interaction could be interpreted as a physical contact between Spc25 and the Cse4 nucleosome. However, this seems unlikely, given our current knowledge of the overall structure of the kinetochore (McAinsh and Marston 2022). Rather, the suppressor mutations in *SPC24*, *SPC25*, and *NDC80* indicate a role for Cse4-R143 in the stability of the centromeric nucleosome. Cse4-R143 lies in the α-N helix of Cse4, which is at the entry–exit site of the DNA that is partially unwrapped in the Cse4 nucleosome compared to a canonical nucleosome (Luger *et al.* 1997). Methylation at this site may enhance unwrapping, thus may destabilize the nucleosome and therefore is expected to counteract centromere function. Similarly, Cse4-K131 is located in the Cenp-A^N^ helix of Cse4, which is wedged between the DNA strands, and methylation at this site may further pry the helices apart and affect nucleosome stability. One possibility is that these PTMs in the core region of the centromeric nucleosome help destabilize the nucleosome during DNA replication and help the replication fork move through the centromeric region. They thus may act in concert with R37 methylation and K49 acetylation in the N-terminus of Cse4, whose abundance increases in the S-phase (Anedchenko *et al.* 2019), concomitantly with the brief dissociation of the kinetochore from centromeric sequences during replication (Kitamura *et al.* 2007).

Of note, both arginine and lysine residues can carry more than one methyl group (symmetric and asymmetric arginine di-methylation and di- and trimethylation of lysine), but the mass spectrometric analysis of Cse4 showed only mono-methylation for R143 and K131. This, however, does not exclude that either residue also exists in higher methylation states in the cell.

We were furthermore surprised that *cse4-R143A* does not show genetic interactions with alleles in other genes encoding NDC80 components. One explanation for this is that the alleles *ndc80-1*, *nuf2-61*, and *spc24-1* may be the result of a kinetochore defect that is mechanistically distinct from *spc25-1*, for instance, a destabilization of the interaction with the microtubule. This cannot be ascertained at this point, because they all carry multiple amino acid substitutions, unlike *spc25-1*, which carries a single exchange, L25P (Nekrasov *et al.* 2003).

It is also noteworthy that *cse4-R143A* caused a defect with *dsn1-7*, because Dsn1 is a direct interaction partner of NDC80c (Hornung *et al.* 2014; Kudalkar *et al.* 2015). A molecular interpretation of this is that the mutation in *DSN1* destabilizes its interaction with the chromatin-proximal side of NDC80c, which causes an enhanced defect upon concomitant destabilization of the centromeric nucleosome by Cse4-R143 mutation.

The observation that R143 and K131 of Cse4 are methylated raises the question which methyltransferases are responsible for the modifications. While this remains to be seen for R143, our genetic analysis of K131 suggests that it is methylated by Set2, which is known for methylation of H3 K36 and K37 (Strahl *et al.* 2002; Santos-Rosa *et al.* 2021). K131 lies within a sequence of Cse4 that shows limited sequence similarity to residues in the N-terminus of H3 surrounding K27 (Fig. 1b). However, H3 K27, a prominent methylation site in higher eukaryotes (Cao *et al.* 2002), is not known to be methylated in *S. cerevisiae*. We speculate that Cse4-K131 represents a non-canonical target of Set2. Notably, the related methyltransferase Set1, whose best-known target residue is H3 K4 (Briggs *et al.* 2001; Roguev *et al.* 2001), also has a function at the kinetochore in that it methylates residues within the Dam1 kinetochore protein, which is part of the Dam1 ring complex that multimerizes around and slides along the microtubules during chromosome segregation (Westermann *et al.* 2006), and this Dam1 methylation counteracts phosphorylation by the Ipl1/Aurora kinase (Zhang *et al.* 2005).

Our discovery of two new PTMs in the core region of the centromeric nucleosome in yeast raises the question whether these modifications are conserved in the CENP-A homologs in higher eukaryotes. So far, several phosphorylation sites, as well as acetylation and ubiquitination, have been described on CENP-A (Fukagawa 2017). Importantly, Cse4-R143, but not Cse4-K131, is conserved in CENP-A (as well as in H3, Supplementary Fig. 5 in Supplementary File 1), and it thus is possible that this residue is also methylated in CENP-A.

## Supplementary Material

iyad028_Supplementary_Data

## Data Availability

Strains and plasmids are available upon request. Supplementary Tables 1 and 2 list the *S. cerevisiae* strains and plasmids used in this study. Sequencing reads were deposited in the National Center for Biotechnology Information (NCBI) Sequence Read Archive (SRA) at http://www.ncbi.nlm.nih.gov/sra under accession no. PRJNA933081. Supplemental material available at GENETICS online.
