# Supplementary material for: Methylation of CENP-A/Cse4 on arginine 143 and lysine 131 regulates kinetochore stability in yeast
Source: Genetics. 2023 Feb 22;223(4):iyad028. doi: 10.1093/genetics/iyad028 (PMC10078908; doi:10.1093/genetics/iyad028)
Supplement: iyad028_Supplementary_Data [file iyad028_supplementary_data.pdf]

Supplementary information

Supplementary Materials and Methods

Mass spectrometric analysis of Cse4

Purification and analysis of 3xHA-tagged Cse4 from yeast cells was performed as described (SAMEL *et al.* 2012). Briefly, partially purified histones from cells expressing 3xHA-Cse4 were separated on 10% SDS-PAGE gels, the Cse4 band excised and digested in-gel with trypsin. Peptide mixtures were separated by nano-liquid chromatography (LC) using an Agilent 1100 Series nanoflow LC system (Agilent Technologies), interfaced to a 7-Tesla LTQ-FT-Ultra mass spectrometer (ThermoFisher Scientific). The LC was operated in one column setup with a 15-cm analytical column (75-µm inner diameter, 350-µm outer diameter) packed with C18 resin (ReproSil, Pur C18AQ 3 µm; Dr. Maisch HPLC GmbH). Solvent A was 0.1% formic acid (FA) and 5% ACN in ddH_2O, and solvent B was 95% ACN with 0.1% FA. Samples were injected in an aqueous 0.1% TFA solution at a flow rate of 500 nL/min. Peptides were separated with a gradient of 0–40% solvent B over 90 min, followed by a gradient of 40–60% for 10 min and 60–80% over 5 min at a flow rate of 250 nL/min. In the LTQ-FT full scan, MS spectra were acquired in a range of m/z 300–1350 by Fourier transform ion cyclotron resonance (FTICR) with resolution R = 100,000 (400 m/z) with a target value of 2.000.000. The five most intense ions were isolated for fragmentation in the linear ion trap using collision-induced dissociation (CID) at a target value of 5.000. Singly charged precursor ions were excluded, and a dynamic exclusion of 60 s was applied. Spray voltage was of 2.4 kV, no sheath and auxiliary gasses were used. Collision gas pressure was 1.3 millitorrs, and normalized collision energy was 35%. Ion selection threshold was 250 counts with an activation q = 0.25. The activation time of 30 ms was applied in MS2 acquisitions. The raw data from LTQ-FT Ultra were analysed by MaxQuant software version 2.0.3.0 (TYANOVA *et al.* 2016). MS/MS spectra were searched against a concatenated forward and reversed version of the yeast ORF database (www.yeastgenome. org). Search parameters were as follows: an initial MS tolerance of 7 ppm, a MS/MS mass tolerance at 0.5 Da, and full trypsin cleavage specificity, allowing for up to 2 missed cleavages. Carbamidomethylation of cysteine was set as a fixed modification, and variable modifications included mono- and dimethylation on lysine and arginine residues, trimethylation on lysines, oxidation on methionine, and acetylation on the N-terminus of proteins. We accepted peptides and proteins with a false discovery rate less than 1%. Filtered data were then manually inspected using the visualization tool integrated in to the MaxQuant suite.

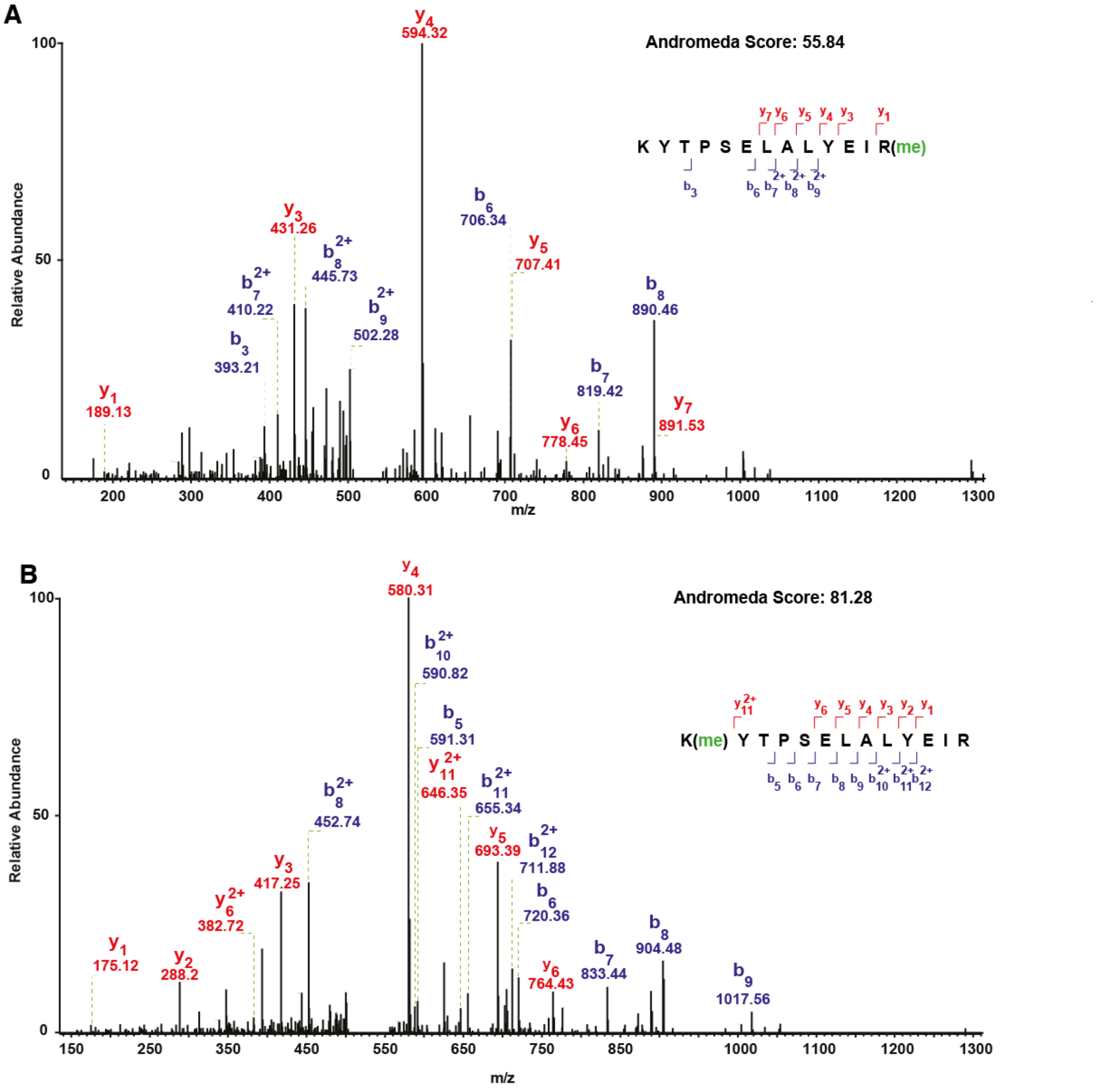

Figure S1: Cse4 is methylated on R143 and K131. A) Collision-induced dissociation analysis and full annotation of the triply charged precursor ion with m/z = 523.2933 and retention time (RT) = 99 min, corresponding to the Cse4 132-KYTPSELALYEIR(me)-143 methylated peptide. B) Collision-induced dissociation analysis and full annotation of the triply charged precursor ion with m/z = 523.2933 and RT= 97 min corresponding to the Cse4 132-K(me)YTPSELALYEIR-143 methylated peptide. In both cases score and expected values as calculated by MaxQuant are reported, and fragments detected in the experiment are indicated in colour (blue for b-ions and red for y-ions).

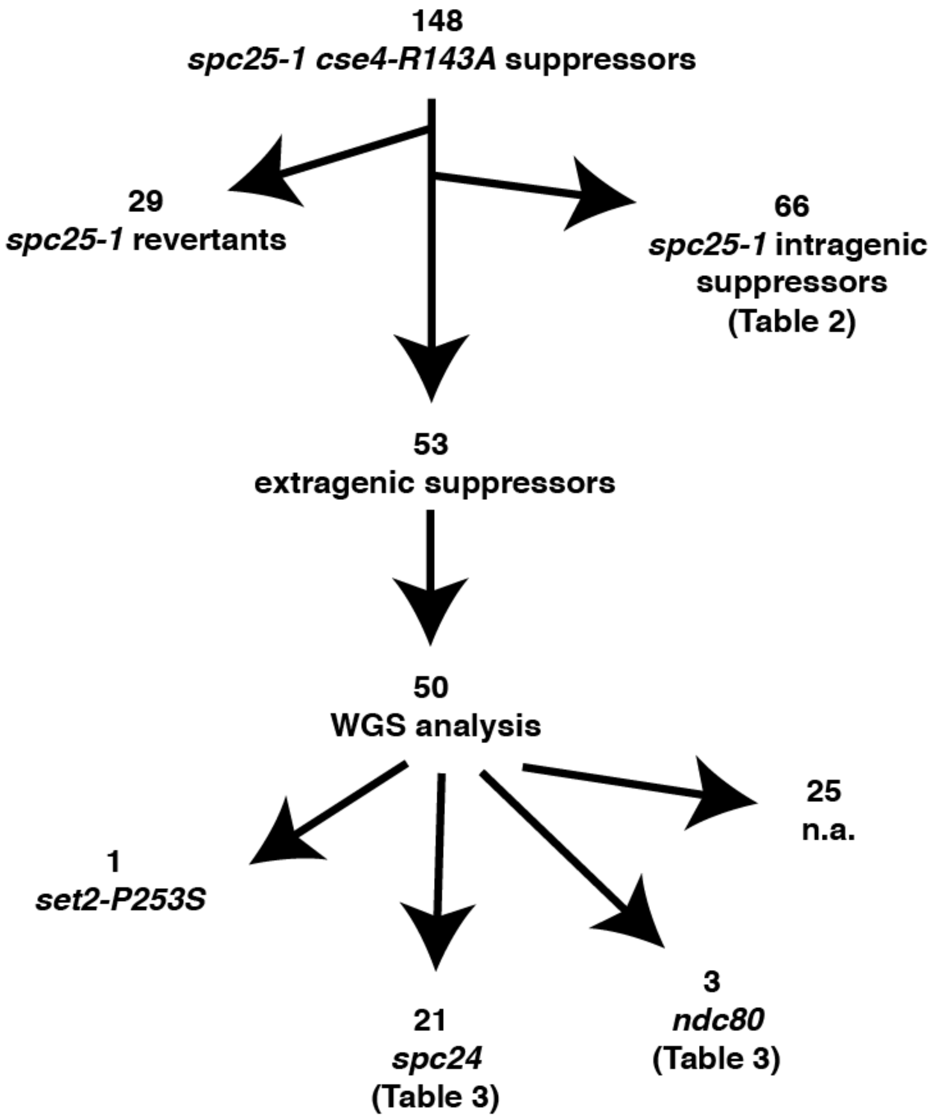

Figure S2: Flow chart showing the analysis of suppressors of the temperature-sensitivity of *spc25-1 cse4-R143A* conducted in this study. Numbers refer to the amount of strains with the respective mutations. 50 strains were subjected to whole-genome sequencing (WGS). The mutation spectrum of *spc24*, *spc25* and *ndc80* alleles is given in the indicated tables.

A

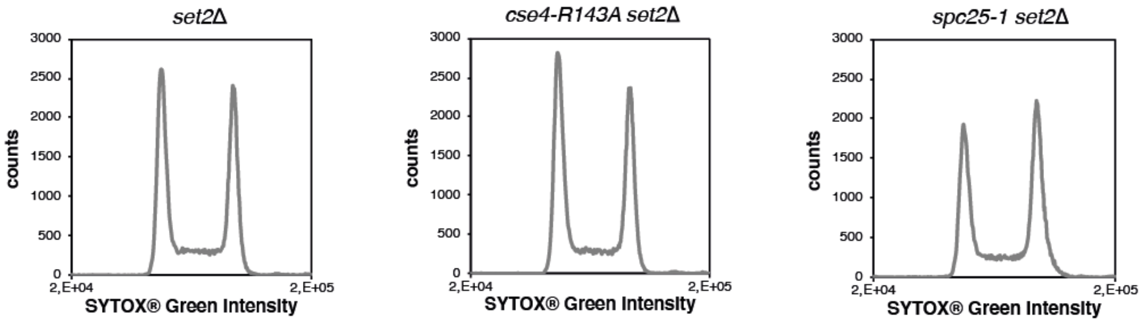

B

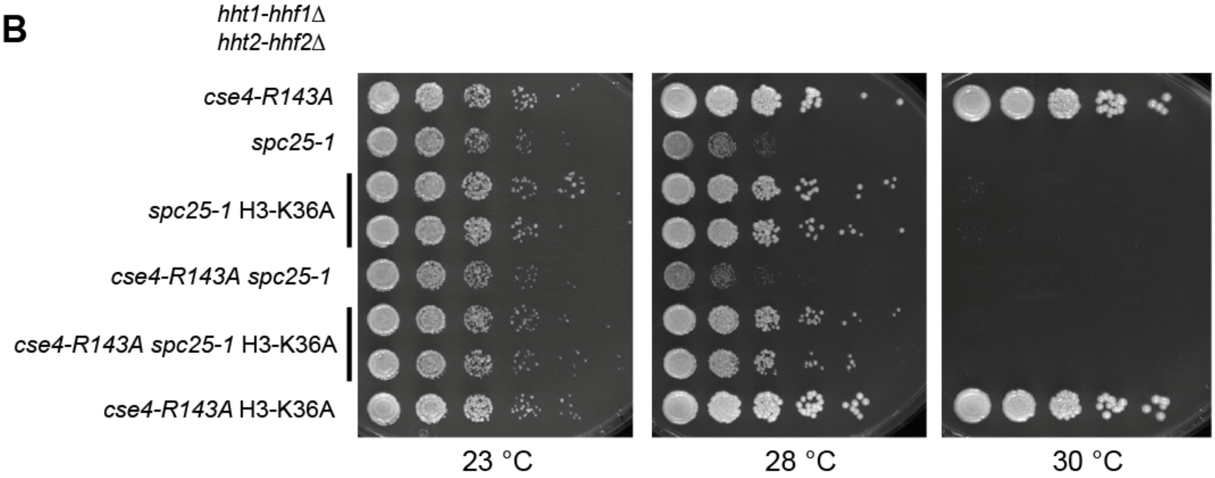

Figure S3: *set2*Δ partially suppresses the growth defect of *spc25-1 cse4-K143A*. A) *set2*Δ does not alter cell-cycle progression in *spc25-1* or *cse4-R143A*. FACS analysis was performed as in Fig. 2C. B) H3-K36A partially suppressed the growth defect of *spc25-1* and *spc25-1 cse4-R143A*. AEY7040, AEY7042 and AEY7045 were transformed with a plasmid carrying H3-K36A/ H4 (pAE3481) or a wild-type control, and the *URA3-HHT1-HHF1* plasmid was removed by counterselection on 5-FOA. Strains were spotted on full medium and incubated for three days at the indicated temperatures.

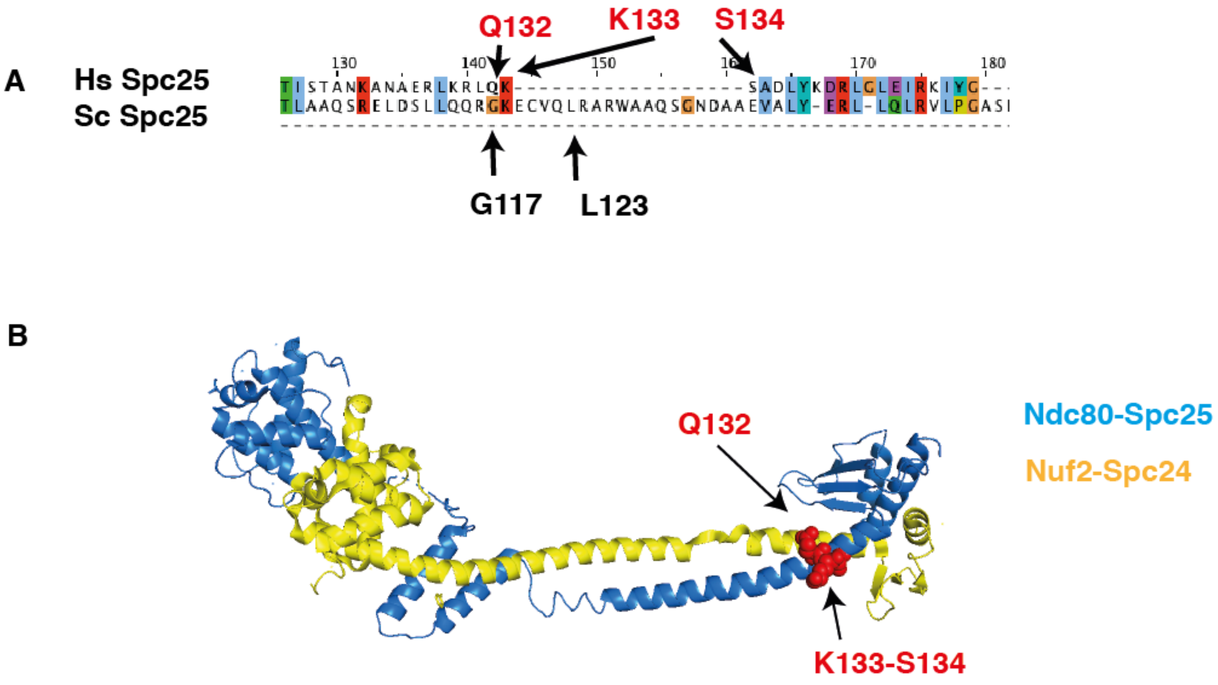

Figure S4: Location of *spc25* suppressor mutations in the context of the Ndc80 $^{\Delta N\text{-bonsai}}$ structure. A) Pairwise alignment of Spc25 from human (Hs) and *S. cerevisiae* (Sc), generated with EMBOSS needle. G117 and L123 mark the suppressor alleles G117D and L123S in Sc Spc25. The equivalent residue to G117 is human Q132. L123 in Sc Spc25 lies in a yeast-specific insert. The residues in human Spc25 flanking the insert are K133 and S134. Numbering above the alignment refers to the position within the alignment. B) The location of Spc25-Q132, -K133 and -S134 in Ndc80 $^{\Delta N\text{-bonsai}}$ is marked in red (PDB 2VE7). Ndc80 $^{\Delta N\text{-bonsai}}$ consists of fusions between Ndc80(1-286) and Spc25(118–224) (blue) and of Nuf2(1–169) and Spc24(122–197) (yellow) (CIFERRI *et al.* 2008).

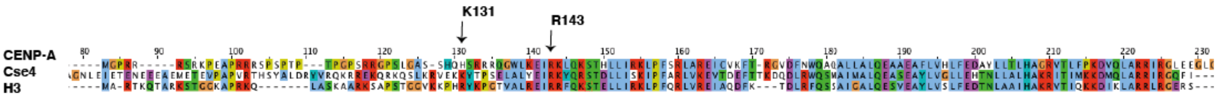

Figure S5: Alignment between Cse4, canonical H3 (*S. cerevisiae*) and CENP-A. The position of Cse4-K131 and -R143 are indicated. Alignment was generated using Clustal Omega.

Table S1: *S. cerevisiae* strains used in this study

Strain number	Genotype	Source *
AEY1	*MATα ade2-101 his3-11,15 trp1-1 leu2-3,112 ura3-1 can1-100*	
AEY2781	*MATα cse4Δ::KanMX ade2-101 his3-11,15 trp1-1 leu2-3,112 ura3-1 can1-100 lys2Δ* + pRS426-3xHA-*CSE4*	
AEY4924	*MAT***a** *ade2-1 trp1-1 can1-100 leu2-3,112 his3-11,15 ura3 ssd1 spc25-1*	John Kilmartin
AEY5064	*MAT***a** *ade2-1 trp1-1 can1-100 leu2-3,112 his3-11,15 ura3 ssd1 spc25-1 cse4Δ::KanMX* + pRS316-3xHA-*CSE4*	
AEY6392	*MAT***a** *ade2-1 trp1-1 can1-100 leu2-3,112 his3-11,15 ura3 dsn1-7*	John Kilmartin
AEY6831	*MATα his3-11,15 trp1-1 leu2-3,112 ura3-1 can1-100 lys2Δ ADE2 cse4-R143A::HisMX*	
AEY6838	*MAT***a** *his3-11,15 trp1-1 leu2-3,112 ura3-1 can1-100 ADE2 LYS2 cse4-R143A::HisMX spc25-1*	
AEY6847	*MAT***a** *ADE2 LYS2, ura3-1 trp1-1 leu2-3 can1-100 ssd1 his3-1 cse4-R143A::HisMX dsn1-7*	
AEY6958	*MAT***a** *his3-11,15 trp1-1 leu2-3,112 ura3-1 can1-100 ADE2 LYS2 cse4-R143A::HisMX spc25-1 ndc80-L681W*	
AEY6974	*MAT***a** *his3-11,15 trp1-1 leu2-3,112 ura3-1 can1-100 ADE2 LYS2 cse4-R143A::HisMX spc25-1 spc24-S45I*	
AEY6975	*MAT***a** *his3-11,15 trp1-1 leu2-3,112 ura3-1 can1-100 ADE2 LYS2 cse4-R143A::HisMX spc25-1 spc24-S59L*	
AEY6977	*MAT***a** *his3-11,15 trp1-1 leu2-3,112 ura3-1 can1-100 ADE2 LYS2 cse4-R143A::HisMX spc25-1 spc24-S59L*	
AEY6978	*MAT***a** *his3-11,15 trp1-1 leu2-3,112 ura3-1 can1-100 ADE2 LYS2 cse4-R143A::HisMX spc25-1 spc24-S59L*	
AEY6994	*MAT***a** *ade2-101 his3-11,15 trp1-1 leu2-3,112 ura3-1 can1-100 cse4-R143A::HisMX spc25-1 set2Δ::KanMX*	
AEY6998	*MAT***a** *ade2-101 his3-11,15 trp1-1 leu2-3,112 ura3-1 can1-100 spc25-1 set2Δ::KanMX*	
AEY7040	*MAT***a** *ADE2 his3-11,15 trp1-1 leu2-3,112 ura3-1 can1-100 hht1-hhf1Δ::LEU2 hht2-hhf2Δ::NatMX cse4-R143A::HisMX spc25-1 + pURA3-HHT1-HHF1*	
AEY7042	*MAT***a** *ADE2 his3-11,15 trp1-1 leu2-3,112 ura3-1 can1-100 hht1-hhf1Δ::LEU2 hht2-hhf2Δ::NatMX spc25-1 + pURA3-HHT1-HHF1*	

AEY7045	*MAT***a** *ADE2 his3-11,15 trp1-1 leu2-3,112 ura3-1 can1-100 hht1-hhf1∆::LEU2 hht2-hhf2∆::NatMX cse4-R143A::HisMX* + *pURA3-HHT1-HHF1*	
AEY7147	*MAT***a** *ade2-101 his3-11,15 trp1-1 leu2-3,112 ura3-1 can1-100 set2∆::KanMX*	Marco Muzi-Falconi

* Unless indicated otherwise, strains were from the laboratory collection or were constructed in the course of this study.

Table S2: Plasmids used in this study

Designation	Plasmid	Source *
pAE264	pRS414	(SIKORSKI AND HIETER 1989)
pAE615	pRS313-3xHA-*CSE4*	(SAMEL *et al.* 2012)
pAE1773	pRS313-3xHA-*cse4-K131A*	
pAE1774	pRS313-3xHA-*cse4-R143A*	
pAE3291	pRS313-3xHA-*cse4-R143E*	
pAE3293	pRS313-3xHA-*cse4-R143Q*	
pAE3481	pRS414-*hht1-K36A-HHF1*	(NAKANISHI *et al.* 2008)
pAE3563	pRS313-3xHA-*cse4-K131A-R143A*	

* Plasmids were from the laboratory collection or constructed in the course of this study, unless indicated otherwise.

Table S3: Mutations that did not show a genetic interaction with *cse4-K131A*

Kinetochore component	Allele tested for genetic interaction with *cse4-K131A*
Cbf1	*cbf1*Δ
CENP-C	*mif2-3*
CBF3	*ctf13-20*
CBF3	*cep3-2*
CBF3	*ndc10-1*
CCAN	*ame1-4*
CCAN	*chl4*Δ
CCAN	*ctf3*Δ
CCAN	*ctf19*Δ
CCAN	*iml3*Δ
CCAN	*mcm21*Δ
CCAN	*okp1-5*
KNL	*spc105-4*
NDC80c	*spc24-1*
NDC80c	*spc25-1*
NDC80c	*ndc80-1*

REFERENCES

Ciferri, C., S. Pasqualato, E. Screpanti, G. Varetti, S. Santaguida *et al.*, 2008 Implications for kinetochore-microtubule attachment from the structure of an engineered Ndc80 complex. Cell 133**:** 427-439.

Nakanishi, S., B. W. Sanderson, K. M. Delventhal, W. D. Bradford, K. Staehling-Hampton *et al.*, 2008 A comprehensive library of histone mutants identifies nucleosomal residues required for H3K4 methylation. Nat Struct Mol Biol 15**:** 881-888.

Samel, A., A. Cuomo, T. Bonaldi and A. E. Ehrenhofer-Murray, 2012 Methylation of CenH3 arginine 37 regulates kinetochore integrity and chromosome segregation. Proc Natl Acad Sci U S A 109**:** 9029-9034.

Sikorski, R. S., and P. Hieter, 1989 A system of shuttle vectors and yeast host strains designed for efficient manipulation of DNA in Saccharomyces cerevisiae. Genetics 122**:** 19-27.

Tyanova, S., T. Temu and J. Cox, 2016 The MaxQuant computational platform for mass spectrometry-based shotgun proteomics. Nat Protoc 11**:** 2301-2319.
